# Microfluidic synthesis of stable and uniform curcumin-loaded solid lipid nanoparticles with high encapsulation efficiency

**DOI:** 10.1039/d4ra08284b

**Published:** 2025-04-04

**Authors:** Seon Tae Kim, Hee Moon Lee, Jae Hwan Jung, Jun-Won Kook

**Affiliations:** a Ajou Energy Science Research Center, Ajou University 206 Worldcup-ro Youngtong-gu Suwon 16499 Republic of Korea kukjw83@ajou.ac.kr; b Intergrative Drug Delivery & Diagnosis Laboratory, Department of Pharmaceutical Engineering, Dankook University 119 Dandae-ro, Dongnam-gu, Cheonan-si Cheonan Chungcheongnam-do 31116 Republic of Korea

## Abstract

Solid Lipid Nanoparticles (SLNs) are a suitable method for encapsulating poorly soluble curcumin by dispersing the drug in solid lipids. However, the commonly used bulk method has disadvantages such as low reproducibility and encapsulation efficiency. To overcome these issues, we used a microfluidic machine to achieve more uniform mixing, resulting in an encapsulation efficiency of over 60%. The synthesized SLNs released over approximately six days and demonstrated colloidal stability for two weeks without aggregation. To synthesize the SLNs, we equipped the microfluidic machine with a temperature controller, which enabled the large-scale production of more reproducible and stable SLNs compared to those synthesized using the existing microfluidic machines.

## Abstract 2

In this study, we developed a nanoformulation with sustained release and improved stability by encapsulating curcumin, a poorly soluble drug, into solid lipid nanoparticles with high encapsulation efficiency using a microfluidic approach.

## Graphical abstract

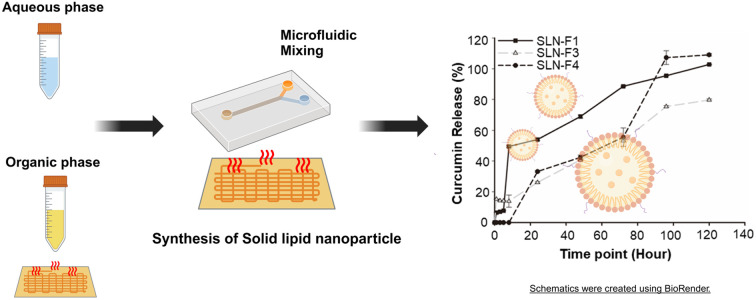

## 1. Introduction

Curcumin (Cur) is a yellow spice found in Indian curry and has a long history as a substance with various pharmacological effects such as antioxidant, anti-inflammatory, dementia prevention, joint protection, and brain health promotion.^1–4^ Cur has been found to be involved in the regulation of biological substances such as TGF-β1/Smad, JNK1/2-ROS, and NF-κB, as well as in anti-inflammatory and antioxidant signaling pathways, thereby exhibiting hepatoprotective, antifibrotic, and anti-fatty liver effects.^5^ Despite these diverse health benefits, Cur has a short half-life in the body, low solubility, rapid digestion, and metabolism, resulting in reduced bioavailability.^6^ Previous studies have introduced nano drug delivery systems (NDDSs) such as nano-emulsions, liposomes, and phospholipid complexes to overcome these limitations.^7,8^ These NDDSs can minimize the toxicity and side effects of the drug delivery by enhancing targeting accuracy.^9^ Nanocarriers synthesized based on NDDSs are generally classified into polymeric nanoparticles and lipid nanoparticles. To achieve low toxicity and targeted sustained release of various active pharmaceutical ingredients (APIs), solid lipid nanoparticles (SLNs) have been introduced.^10,11^ SLNs are composed of a hydrophobic core and a hydrophilic outer layer, allowing the encapsulation of both hydrophilic and hydrophobic molecules.^12^ When the core material is a hydrophobic APIs, the core content correlated with APIs release. SLNs act as a physicochemical barrier to encapsulated APIs. Unlike lipid nanoparticles, they offer greater stability and resistance compared to other lipid formulations and polymer systems.^13,14^ In particular, the use of solid lipids reduces the mobility of the APIs, thereby protecting the APIs and increasing the encapsulation efficiency.^15,16^ The most important factor in the process of manufacturing SLNs containing APIs is the reproducibility of a simple and uniform morphology. One of the proposed methods to achieve this is microfluidics. This method allows for the production of nanoparticles with uniform size through a continuous process in a short amount of time,^17,18^ enabling a wide range of applications including lipid-based formulations,^19,20^ metal colloids,^21,22^ and polymers. Using microfluidics allows for the control of size, shape, and encapsulation efficiency of SLNs; a problem arises with the wax-like core material used in SLNs, which crystallizes at lower temperatures, thereby limiting the APIs loading capacity.^23,24^ Cetyl palmitate is a substance used as a lipid component in SLNs that remains solid at room temperature but becomes liquid above a certain temperature.^25^ When SLNs are manufactured using microfluidics at these elevated temperatures, it is expected not only to improve the encapsulation efficiency of the APIs but also to enhance the homogeneity.^26^

When using microfluidic technology for nanoparticle synthesis, several strategies can be employed to increase encapsulation efficiency if it is initially low: first, adjusting the flow rates of the dispersed and continuous phases can help control the size and uniformity of the nanoparticles, leading to better encapsulation efficiency. Second, altering the concentration and type of surfactants, lipids, or polymers can enhance the stability of the nanoparticles and improve encapsulation efficiency. Third, controlling the temperature during the synthesis process can prevent premature crystallization of lipids and ensure proper encapsulation of the APIs.^27,28^ Using microfluidics, a low polydispersity index (PDI) indicates a uniform size distribution of nanoparticles.^29^ The advantages of having a low PDI include improved stability, consistent release profiles, enhanced targeting, reduced side effects, and reproducibility. Uniform nanoparticles are more stable in suspension, reducing the risk of aggregation over time.^30^ Homogeneous nanoparticles provide more predictable and consistent drug release profiles, enhancing therapeutic efficacy.^31^ With a uniform size, nanoparticles can more effectively target specific tissues or cells, improving the precision of drug delivery. Consistent particle size reduces the likelihood of non-specific distribution and side effects, leading to a safer treatment profile.^32^ Low PDI ensures that the nanoparticles synthesis process is reproducible which is critical for large-scale manufacturing and clinical applications.^19^ Unlike conventional methods of manufacturing SLNs using microfluidics, this approach differentiates itself by using a film heater during the SLN manufacturing process. This allows for the stable production of SLNs with high melting point solid lipids, ensuring high encapsulation efficiency and reproducibility for Cur-loaded SLNs (Cur-SLNs).

## 2. Materials & methods

### 2.1 Materials

All chemical substances were used as received without further purification or distillation. 1,2-Distearoyl-*sn-glycero*-3-phosphoethanolamine-*N*-[methoxy(polyethylene glycol)-2000] (ammonium salt) (C18-PEG-2000) was purchased from Avanti Polar Lipids (USA). Cetyl palmitate, Cur, and Tween 20 (TW20) were purchased from Sigma Aldrich (USA). Tween 80 (TW80) was Purchased from Duksan Science (Korea), and polyvinyl alcohol 500(PVA) was purchased from Daejung Chemicals & Metals (Korea). All materials were of analytical grade. All aqueous solutions were prepared using purified water obtained with LabDUO 200 (BaLmann Tech, Korea). Opti-MEM™ Media was purchased from Thermo Fisher Scientific (USA). Phosphate buffer saline (10× PBS) was purchased from Dyne Bio (Korea).

### 2.2 Preparation of Cur-SLNs using a microfluidic machine

The microfluidic machine (NanoKRAShot™ OPTI, KRON, Korea) was modified to enable temperature control. To construct the temperature control apparatus, a temperature controller and sensor (Scipia, Korea) were integrated with a Kapton film heater (Device-mart, Korea). The film heater was affixed to the cylinder containing the organic phase and the backside of the microfluidic chip (NanoKRAChip™ MON, KRON, Korea) in the microfluidic device, secured using 3M tape.

For the synthesis of Cur-SLNs, the organic phase comprised 10 mg mL^−1^ of cetyl palmitate, 3 mg mL^−1^ of C18-PEG 2000, and 3, 5, or 7 mg mL^−1^ of Cur dissolved in 1 mL of ethanol. The aqueous phase was prepared by dissolving TW20, TW80, and PVA in deionized water (DI) at varying concentrations. To facilitate the rapid adsorption of the surfactant onto the solid lipid surface and promote the formation of uniform nanoparticles, a flow rate of 10 mL min^−1^ was applied. To investigate the impact of the organic-to-aqueous (O/W) phase mixing ratio on particle formation, the organic and aqueous phases were mixed at ratios of 1 : 3 and 1 : 5. The resulting samples underwent centrifugal separation using an Amicon Ultra Centrifugal Filter (10 kDa, 100 kDa Thermo Fisher, USA) at 4 °C, 10 000 rpm for 15 minutes to remove unencapsulated drug and residual surfactants. Subsequently, the samples were redispersed in deionized water (repeated 4 times) and stored at 4 °C.

### 2.3 Characterization of Cur-SLNs

The particle size distribution of the synthesized SLNs was characterized using dynamic light scattering (DLS) analysis, performed with a Zetasizer Pro (Malvern Instruments, UK). A 1 mL aliquot of the particle suspension was diluted 20-fold in PBS, placed in a folded capillary cell (DTS 1070, Malvern Instruments, UK), and measured at 25 °C. Each experiment was conducted in triplicate, with the results expressed as the mean and standard deviation of three independent measurements. The morphology of Cur-SLNs were observed by Cs-corrector transmission electron microscope (Cs-TEM, JEM-ARM200F, JEOL, Japan). The samples for Cs-TEM analyses were prepared by mounting diluted onto a grid.

For the encapsulation efficiency (EE%) and content analysis of Cur, a hydrophobic drug, measurements were performed using a Microplate Reader (Synergy HTX, BioTek, USA) by measuring absorbance at 425 nm. Cur was dissolved in ethanol to prepare standard solutions with concentrations ranging from 0.5 μg mL^−1^ to 500 μg mL^−1^, which were used to generate a standard curve. This standard curve was then utilized to quantify the amount of encapsulated Cur within the SLNs.
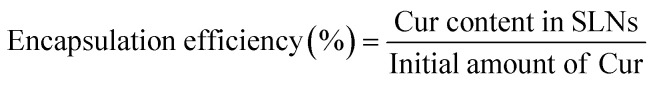


### 2.4 *In Vitro* release test

The release test for Cur from the SLNs was conducted using a dialysis cup (Slide-A-Lyzer™ Mini Dialysis Units, 10 kDa, Thermo Fisher, USA). A 200 μL aliquot of the SLN suspension was diluted 1 : 1 with cell culture media and then placed into the dialysis cup. The receptor solution, consisting of 10 mL of PBS with 1% w/v TW20, was continuously stirred at 500 rpm and maintained at 37 °C. At designated time points over a 120 hours period, 1 mL of the receptor solution was collected, and an equivalent volume of fresh PBS with 1% w/v TW20 was added. The collected samples were analyzed for Cur content by measuring absorbance at 425 nm using a microplate reader. Each experiment was conducted in triplicate, with results reported as the mean and standard deviation of three independent measurements.

### 2.5 Stability test

The stability of the SLNs was assessed by analyzing their size distribution in PBS (pH 7.4) over a 14 days period. For this test, 200 μL of the SLN suspension was incubated in 2.8 mL of the respective solution at 37 °C. To evaluate the change in particle size over time, samples were collected at predetermined intervals and diluted 20-fold in PBS (pH 7.4), and subsequently analyzed using DLS.

### 2.6 FT-IR and DSC analysis

Attenuated total reflectance Fourier transform infrared (ATR-FTIR) spectroscopy was performed on Cur, Cur-SLNs in the wavenumber range of 600–4000 cm^−1^ at room temperature (Thermo Scientific™, Nicolet iS50). Differential Scanning Calorimetry (DSC) was conducted using standard aluminum pans. A temperature range of 0–200 °C was employed for all samples and formulations, with a heating rate of 10 °C min^−1^.

### 2.7 Statistical analysis

All experiments were conducted in triplicate, and the results are presented as the mean ± standard deviation (SD). A one-way analysis of variance (ANOVA) was performed to evaluate statistical differences among groups. A *p*-value of less than 0.05 was considered statistically significant.

## 3. Results and discussion

### 3.1 Preparation and characterization of Cur-SLNs

Conventional bulk methods for SLN synthesis are time-consuming and suffer poor reproducibility and high polydispersity due to difficulties in achieving uniform mixing.^33^ To address these issues, we utilized a microfluidic device for SLN synthesis. Microfluidic devices ensure high encapsulation efficiency and PDI by facilitating uniform mixing through microfluidic flow.^34^ Furthermore, the high reproducibility characteristic of microfluidic systems allows for scalability from laboratory to practical application.

**Fig. 1:**
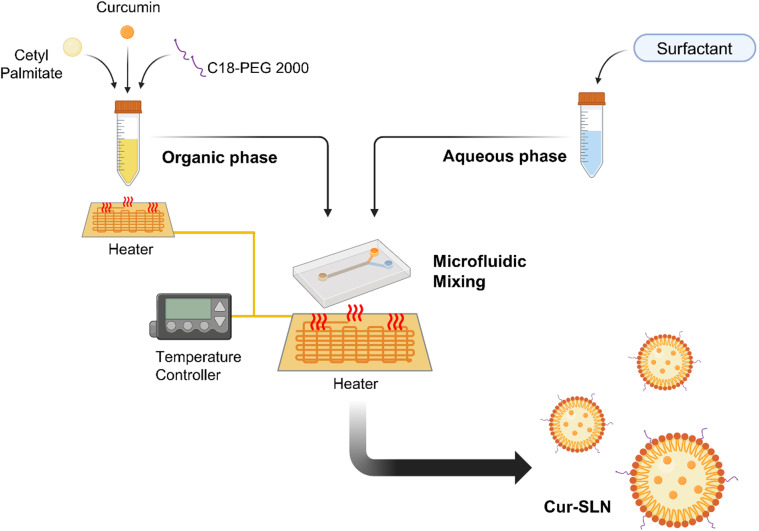
Schematic illustration of the synthesis of Cur-SLNs using a microfluidic system integrated with a heating system to enhance encapsulation efficiency and achieve greater particle homogeneity. Schematics were created using BioRender.

Cetyl palmitate, which has low biotoxicity, a high melting point, and excellent particle stability, was selected as a solid lipid.^35^ A critical consideration in producing SLNs with a microfluidic device is temperature control. Cetyl palmitate, the solid lipid used, has a melting point of approximately 55 °C and remains solid below this temperature.^26^ Maintaining cetyl palmitate in its liquid form is essential for nanoparticle synthesis. To achieve this, we integrated a temperature control device into the microfluidic system (Fig. 1). The organic phase reservoir and sample cylinder were equipped with a film heater, and a heater was also attached to the microfluidic chip where mixing occurs, maintaining the system at approximately 60 °C. This setup ensured homogeneous mixing of the solid lipids without disrupting the microfluidic flow.

SLNs were formulated with a core matrix of cetyl palmitate encapsulating Cur, while surfactants were employed to stabilize the surface of Cur-SLNs. Additionally, C18-PEG-2000 was incorporated into the lipid matrix to prevent aggregation and improve the dispersion stability of Cur-SLNs in aqueous environments. Several factors influence the characteristics of SLNs during synthesis, including the type and concentration of surfactant, the lipid composition, and the O/W mixing ratio. To optimize synthesis conditions three surfactants, TW20, TW80, and PVA were used to prepare aqueous phases. The resulting particle characteristics were systematically analyzed (Table 1). The concentration of Cur in the organic phase was standardized at 5 mg mL^−1^, based on its maximum solubility of 10 mg mL^−1^ in ethanol. For the initial screening of surfactant types, the surfactant concentration was maintained at 2% w/v. The characteristics of the resultant particles were then examined at the O/W mixing ratios of 1 : 3 and 1 : 5. Additionally, to enhance the cellular internalization of the synthesized SLNs, we aimed to produce particles with an approximate size of 100 nm.^36^

**Table 1:** Characteristics of SLNs prepared with different surfactants and oil-to-water (O/W) mixing ratio. The concentration of Cur was maintained at 5 mg mL^−1^, while the surfactant concentration was kept constant at 2% w/v during SLN synthesis

Surfactant, mixing ratio (O/W)	MW (Da)	HLB value	Size (nm)	PDI	Zeta potential (mV)
TW20, 1 : 3	1228	16.7	137.3 ± 4.4	0.13 ± 0.02	−2.69 ± 0.03
TW20, 1 : 5	1228	16.7	92.3 ± 5.2	0.27 ± 0.02	−3.95 ± 0.50
TW80, 1 : 3	1310	15.0	100.6 ± 12.8	0.36 ± 0.02	−0.81 ± 0.14
TW80, 1 : 5	1310	15.0	89.9 ± 4.8	0.42 ± 0.02	−1.79 ± 1.30
PVA, 1 : 3	22 000	18.0	573.7 ± 89.7	0.62 ± 0.09	−23.57 ± 0.01
PVA, 1 : 5	22 000	18.0	801.5 ± 364.5	1.08 ± 0.83	−22.31 ± 3.18

SLNs utilizing TW20 as a surfactant exhibited a particle size of 137.3 ± 4.4 nm, with a PDI of 0.13 ± 0.02 and a zeta potential of −2.69 ± 0.03 mV at the O/W mixing ratio of 1 : 3, and a particle size was 92.3 ± 5.2 nm, with a PDI of 0.27 ± 0.02 and a zeta potential of −3.95 ± 0.50 mV at the O/W mixing ratio of 1 : 5. On the other hand, SLNs employing TW80 as a surfactant demonstrated a particle size of 100.6 ± 12.8 nm (PDI 0.36 ± 0.02) and a zeta potential of −0.81 ± 0.14 mV at the mixing ratio of 1 : 3, and a particle size of 89.9 ± 4.8 nm (PDI 0.42 ± 0.02) and zeta potential of −1.79 ± 1.30 mV at the mixing ratio of 1 : 5. For PVA, at the mixing ratio of 1 : 3, a particle size was measured to be approximately 573.7 ± 89.7 nm (PDI 0.62 ± 0.09) and a zeta potential of −23.57 ± 0.01 mV. At the mixing ratio 1 : 5, a particle size was 801.5 ± 364.5 nm (PDI was 1.08 ± 0.83) and a zeta potential of −22.31 ± 3.18 mV.

**Fig. 2:**
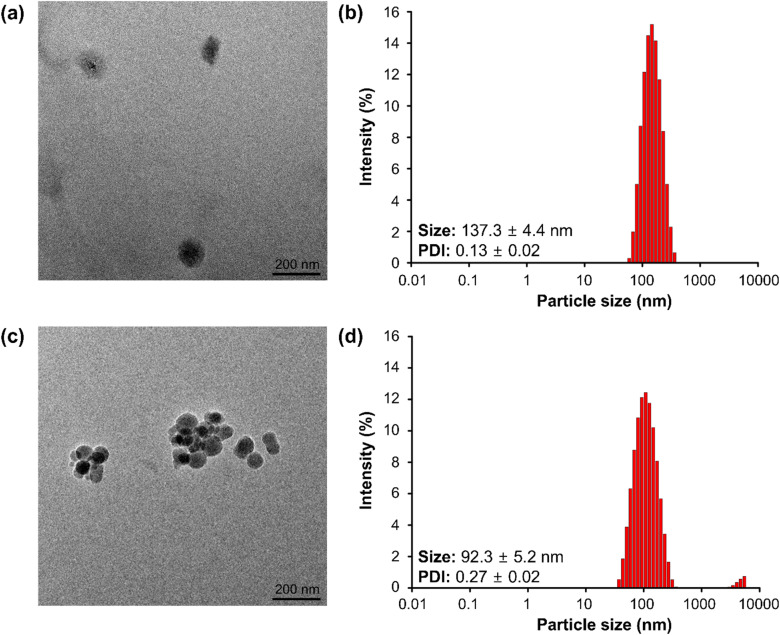
Analysis of SLN characteristics as a function of O/W mixing ratio variations. TEM image (a) and DLS measurement result (b) of SLNs at an O/W mixing ratio of 1 : 3. TEM image (c) and DLS measurement result (d) of SLNs at an O/W mixing ratio of 1 : 5 (with 2% TW 20, 5 mg mL^−1^ Cur).

As the molecular weight of the surfactant decreased, a corresponding reduction in the PDI was observed, indicating an increase in particle homogeneity. Specifically, using TW20 with a molecular weight (MW) of 1228 Da resulted in a PDI value of less than 0.3, signifying a more uniform particle distribution. In contrast, surfactants with higher molecular weights, such as TW80 (MW = 1310 Da) and PVA (MW = 22 000 Da), exhibited elevated PDI values exceeding 0.3, reflecting more significant heterogeneity. Furthermore, the synthesized SLNs were significantly smaller when TW20 and TW80 were employed than PVA. This phenomenon can be attributed to the faster adsorption of low-molecular-weight surfactants onto the lipid surface, facilitating the formation of more uniform and smaller particles.^37^ The alteration in the O/W mixing ratio significantly influenced the size of SLNs. Specifically, increasing the aqueous phase ratio from 1 : 3 to 1 : 5 reduced SLN size (TW 20), as confirmed by transmission electron microscopy (TEM) analysis and the particle size distribution profile obtained from dynamic light scattering (DLS) measurements (Fig. 2). The aggregation of some particles observed in the TEM images appears to be caused by the “coffee-ring” effect during the sample drying process.^38^ However, the DLS measurement results for the sample confirmed that it had uniform particle size. When only the O/W mixing ratio was adjusted to 1 : 3 and 1 : 5 under identical conditions (5 mg mL^−1^ curcumin, 2% Tween 20), the SLN sizes measured by DLS were 137.3 ± 4.4 nm for the 1 : 3 ratio and 92.3 ± 5.2 nm for the 1 : 5 ratio (Table 2).

**Table 2:** Comparison of SLN characteristics based on TW20 concentration and O/W mixing ratio. The concentration of Cur was maintained at 5 mg mL^−1^ during SLN synthesis

TW20 (w/v), mixing ratio (O/W)	Size (nm)	PDI	Zeta potential (mV)
1%, 1 : 3	1317 ± 209.7	0.76 ± 0.04	−2.94 ± 0.37
1%, 1 : 5	142.3 ± 104.4	0.33 ± 0.08	−1.96 ± 1.11
2%, 1 : 3	137.3 ± 4.4	0.13 ± 0.02	−2.69 ± 0.02
2%, 1 : 5	92.3 ± 5.2	0.27 ± 0.02	−3.95 ± 0.50
3%, 1 : 3	132 ± 3.4	0.36 ± 0.04	−2.00 ± 1.24
3%, 1 : 5	81.5 ± 5.0	0.40 ± 0.04	−0.66 ± 1.61

The SLNs synthesized using microfluidic technology exhibited a smoother and more spherical morphology compared to those produced *via* the conventional bulk method.^33^ This size reduction is attributed to the increased surfactant concentration relative to the aqueous phase, which likely led to decreased interfacial tension, ultimately reducing the particle size.^39^

The Hydrophile–Lipophile Balance (HLB) value significantly influenced the characteristics of SLNs. Notably, the smallest SLN size was achieved using TW80, which possesses a lower HLB value. This effect is attributed to the enhanced affinity of lower HLB surfactants for lipophilic drugs, which facilitates more rapid binding. This rapid interaction reduces interfacial tension between the O/W phases, thereby decreasing particle size.^40^ The synthesis of SLNs involves intricate interactions between various components. Based on the experimental data, TW20 was identified as the optimal surfactant for Cur-SLNs due to its favorable particle size distribution and PDI.

To examine the impact of surfactant concentration on the physicochemical properties of SLNs, which were synthesized with TW20 as the surfactant at O/W mixing ratios of 1 : 3 and 1 : 5 and at surfactant concentrations of 1, 2, and 3% (w/v). As with surfactant screening, the organic phase concentration of curcumin was set to 5 mg mL^−1^ (Table 2). At a 2% (w/v) surfactant concentration, SLNs prepared with the 1 : 3 mixing ratio demonstrated a PDI of 0.13 ± 0.02, whereas those with the 1 : 5 mixing ratio exhibited a PDI of 0.27 ± 0.02.

**Fig. 3:**
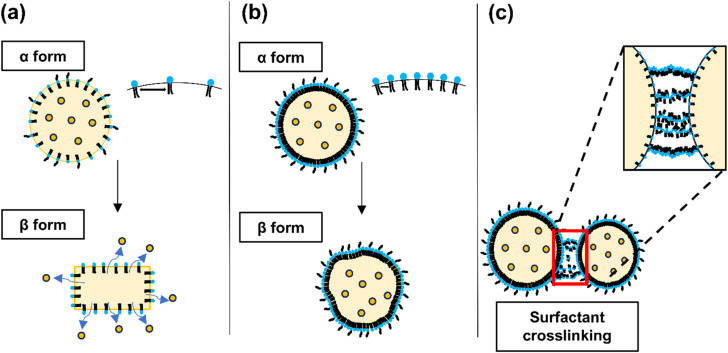
Schematic representation illustrating the changes in SLN crystallization based on surfactant concentration. The figure depicts the effects of (a) low, (b) medium, and (c) high surfactant concentrations on the structural integrity and deformation of SLNs. The diagrams are illustrative and represent possible configurations of SLNs at varying surfactant levels.

In contrast, at surfactant concentrations of 1% and 3% (w/v), the PDI values for both mixing ratios were observed to exceed 0.3. This phenomenon can be attributed to the crystallization behavior of solid lipids (Fig. 3). Initially, solid lipids tend to crystallize into a metastable α-form, characterized by a spherical morphology, which transitions into a more thermodynamically stable β-form with an elongated structure over time. Surfactants are crucial in stabilizing this system by shielding the hydrophobic lipid chains, inhibiting the conformational transition from the α- to β-form. Consequently, this prevents aggregation and gelation between SLNs.^41^

When the surfactant-to-lipid ratio is suboptimal, a phase transition in the lipid crystal structure is observed, leading to increased polydispersity and particle aggregation. This phenomenon is evidenced by the elevated particle size and PDI at low surfactant concentrations (1%) and low O/W mixing ratios (1 : 3). The kinetics of lipid-surfactant interactions accelerate with increasing surfactant concentrations. These findings suggest that SLNs with reduced particle size and low polydispersity can be achieved by optimizing the surfactant concentration and lipid-surfactant mixing ratio.

On the other hand, the formation of cross-links between nanoparticles at elevated surfactant concentrations has been observed to increase cytotoxicity.^42^ This phenomenon is corroborated by the increase in PDI at higher surfactant-to-nanoparticle mixing ratios, suggesting enhanced nanoparticle cross-linking. Compared to conventional methods, the rapid mixing facilitated by microfluidic flow further accentuates differences in particle size and PDI as a function of the mixing ratio at a constant surfactant concentration. Therefore, optimizing the surfactant concentration relative to the drug and lipid concentrations is crucial to achieving the desired nanoparticle characteristics. Our results indicated that using 2% w/v TW20 in the aqueous phase is optimal for the preparation of SLNs, effectively balancing particle size and polydispersity.

Enhanced encapsulation efficiency is crucial for improving therapeutic efficacy, as it allows for higher drug loading within a fixed formulation volume. To optimize encapsulation efficiency, SLNs were synthesized by varying the concentration of Cur in the organic phase (Table 3). SLNs were prepared in the organic phase containing Cur at a concentrations of 3 mg mL^−1^, with O/W mixing ratios of 1 : 3 (SLN-F1) and 1 : 5 (SLN-F2). These formulations achieved encapsulation efficiencies of 71.33 ± 1.00% and 53.66 ± 1.38%, respectively with corresponding Cur concentrations within the SLN formulations of 535.0 ± 7.5 μg mL^−1^ and 268.3 ± 6.9 μg mL^−1^. Upon increasing the Cur concentration to 5 mg mL^−1^, SLNs prepared at the same mixing ratios (1 : 3 for SLN-F3 and 1 : 5 for SLN-F4) exhibited higher encapsulation efficiencies, reaching 76.05 ± 1.73% and 65.20 ± 1.38%, respectively. The corresponding Cur concentrations in these formulations were 950.6 ± 21.6 μg mL^−1^ and 543.3 ± 11.5 μg mL^−1^.

**Table 3:** Comparison of SLN characteristics based on Cur concentrations and O/W mixing ratio. The TW20 concentration was maintained at 2% w/v during SLN synthesis

Sample name	Cur in organic phase (mg mL^−1^)	Mixing ratio(O/W)	Size (nm)	PDI	Encapsulation efficiency (EE%)	Cur conc. (μg mL^−1^)
SLN-F1	3	1 : 3	158.4 ± 1.7	0.20 ± 0.01	71.33 ± 1.00	535.0 ± 7.5
SLN-F2	3	1 : 5	180.0 ± 4.5	0.16 ± 0.02	53.66 ± 1.38	268.3 ± 6.9
SLN-F3	5	1 : 3	137.3 ± 4.4	0.13 ± 0.02	76.05 ± 1.73	950.6 ± 21.6
SLN-F4	5	1 : 5	92.3 ± 5.2	0.27 ± 0.02	65.20 ± 1.38	543.3 ± 11.5
SLN-F5	7	1 : 3	835.3 ± 54.9	0.60 ± 0.08	23.62 ± 0.24	413.4 ± 4.2
SLN-F6	7	1 : 5	177.7 ± 0.6	0.15 ± 0.00	35.42 ± 0.35	413.2 ± 4.1

However, at the highest Cur concentration of 7 mg mL^−1^, both mixing ratios (1 : 3 for SLN-F5 and 1 : 5 for SLN-F6) resulted in a marked decrease in encapsulation efficiency. Specifically, the encapsulation efficiencies dropped to 23.62 ± 0.24% and 35.42 ± 0.35% for the 1 : 3 and 1 : 5 mixing ratios, respectively, with corresponding Cur concentrations of 413.4 ± 4.2 μg mL^−1^ and 413.2 ± 4.1 μg mL^−1^. It is hypothesized that the excess Cur not encapsulated into SLN did not dissolve in the aqueous medium, leading to its precipitation and subsequently affecting nanoparticle synthesis. Except for the 7 mg mL^−1^ Cur, the encapsulation efficiency decreased as the volume ratio of the aqueous phase increased. This phenomenon can be attributed to the increased volume of the aqueous phase, which likely reduced the amount of drug encapsulated into the organic phase.^39^ Consequently, we optimized the aqueous phase conditions using 2% w/v TW20, which produced appropriate particle size and polydispersity. Furthermore, we synthesized Cur-SLNs using 3 mg mL^−1^ Cur at O/W 1 : 3 mixing ratio (SLN-F1) and 5 mg mL^−1^ Cur at the 1 : 3 and 1 : 5 mixing ratios (SLN-F3 and SLN-F4), which showed more than 60% encapsulation efficiency.

In previous studies, the encapsulation efficiency of Cur in SLNs manufactured using the bulk method was reported to be approximately 40%. However, in this study, SLNs synthesized using the microfluidic method achieved an encapsulation efficiency of up to 76%, nearly doubling the drug entrapment rate.^33^ This improvement is attributed to the microfluidic chip's structure, which facilitates uniform fluid mixing, thereby enhancing drug loading within the solid lipid matrix.^34^

### 3.2 Analysis of the chemical properties of Cur-SLNs

**Fig. 4:**
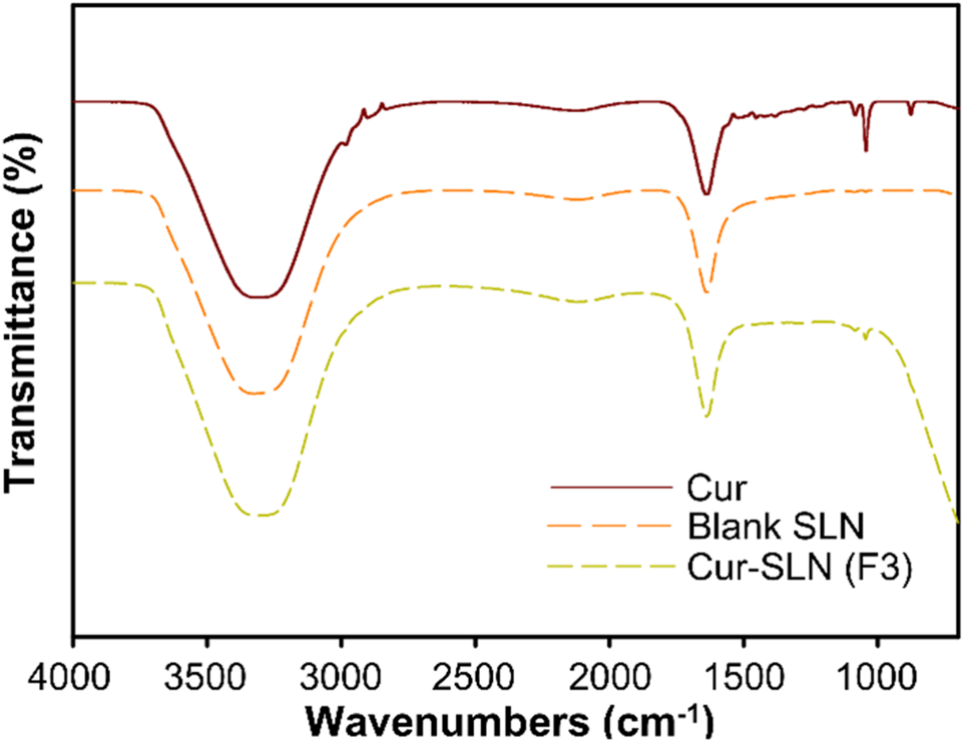
FT-IR spectra of Cur, Cur-SLN (SLN-F3), and blank SLN.

In ATR-FTIR spectrum of Cur, Cur-SLNs (SLN-F3), and blank SLNs was shown in Fig. 4, in which Cur showed a number of characteristic bands. To confirm the successful encapsulation of Cur within SLNs, SLN-F3, which exhibited the highest encapsulation efficiency, was analyzed using FT-IR. Among these, the absorption peak at 3312 cm^−1^ corresponds to hydrogen bonding (–OH) stretching and can correspond to the conjugated carbonyl group (C=O) at 1637 cm^−1^.^43^ There were no shifts or losses of peaks in the ATR-FTIR spectrum for both blank SLN and Cur-SLNs, which means that Cur is well compatible with other components used in the SLN formulation synthesis process. By observing the absorption peaks of Cur and Cur-SLNs, an absorption peak is detected at 937 cm^−1^ due to the benzoate trans –CH vibration, which indicates that Cur is well loaded into the Cur-SLNs. In addition, the identification of hydrogen bonding and the conjugated carbonyl group suggests that Cur is well encapsulated within the SLN.^44^

### 3.3 Solid state of Cur-SLNs

**Fig. 5:**
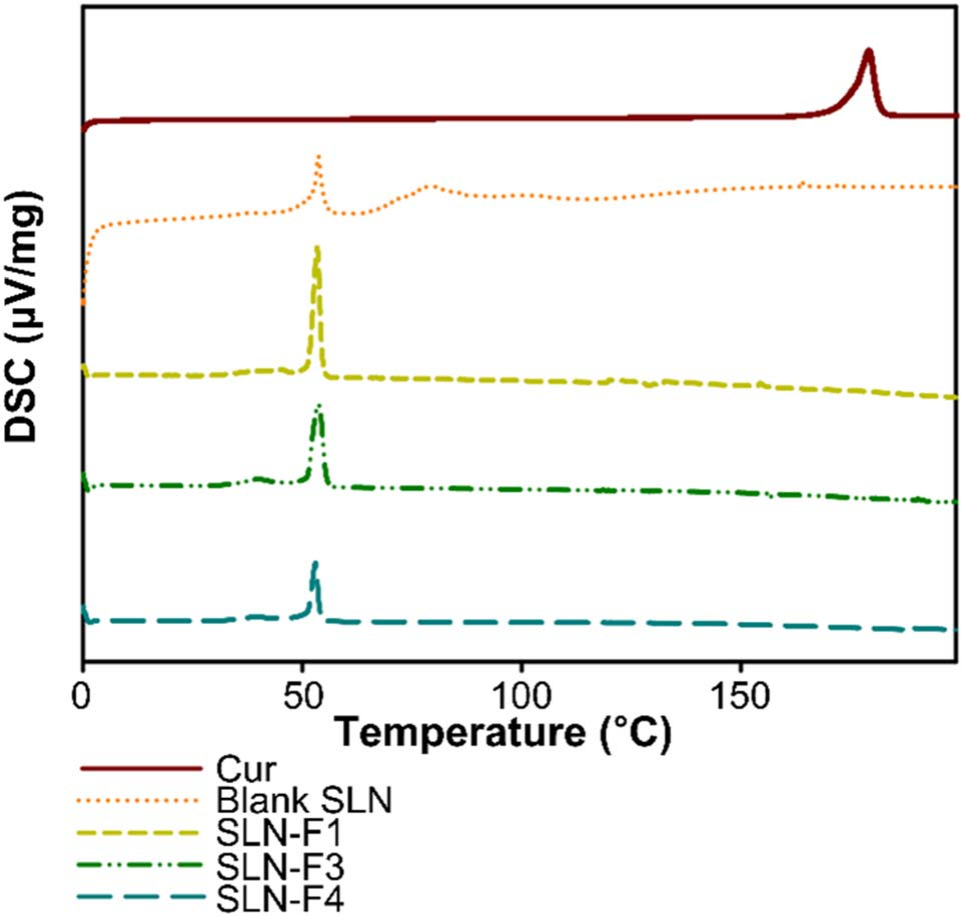
DSC thermogram graphs of Cur, Blank SLN without Cur, SLN-F1, SLN-F3, and SLN-F4.

In Cur-SLNs, the nanoparticles must remain in a solid state to be used as a drug delivery system. To verify this and evaluate the lipid crystallinity of the SLNs, DSC analysis was conducted on pure Cur, Cur-SLNs, and blank SLNs. The DSC thermograms are shown in Fig. 5. The DSC thermogram of pure Cur shows a sharp peak at 179.24 °C. The melting point is measured slightly higher than the known temperature (173.1 °C), which is believed to be due to the presence of Cur in both keto and enol forms.^45^ In Fig. 5, an endothermic peak of Cur-SLNs appears at around 53 °C, indicating the melting of the solid SLNs. This phenomenon is one of the most important characteristics of the storage stability and sustained drug release properties of Cur-SLNs. Furthermore, in the thermograms of SLN-F1, SLN-F3, and SLN-F4, no melting peak of Cur is observed, which indicates that Cur is well encapsulated with higher enthalpy value in the samples with Cur encapsulation compared to blank SLNs. The enthalpy value of SLN-F1 was 53.32 J g^−1^, SLN-F3 was 53.58 J g^−1^, SLN-F4 was 52.88 J g^−1^, and the enthalpy value of Blank SLN was 45.34 J g^−1^. The mixing ratio of Blank SLN was 1 : 3. In other words, the SLN molecules exist in a more ordered state with increased enthalpy and homogeneity of the Cur-SLNs achieved through the microfluidic machine under temperature-controlled conditions.

### 3.4 *In vitro* Cur release test

**Fig. 6:**
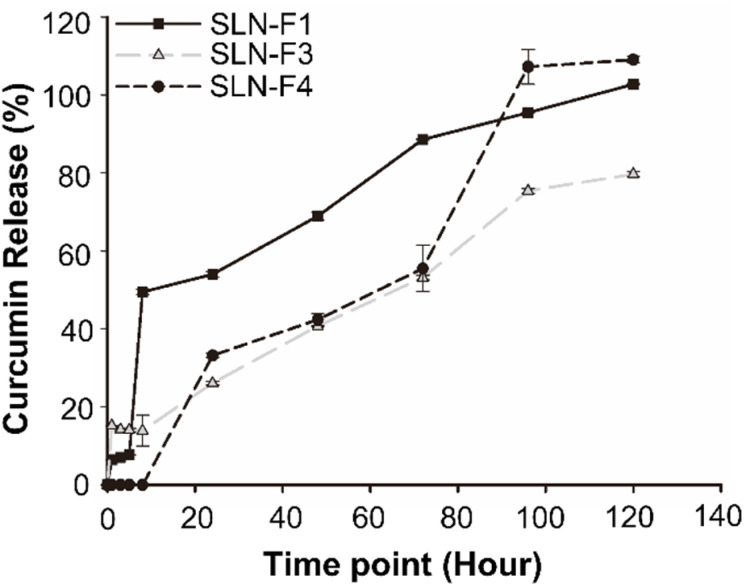
*In vitro* release profiles of Cur from SLN in medium at 37 °C. 1% w/v TW20, PBS were used receptor compartment. Results are reported as mean ± SD, *n* = 3.

*In vitro* drug release studies of SLNs were performed using the dialysis membrane method to simulate *in vivo* conditions (Fig. 6). The SLNs were dispersed in Opti-MEM medium and placed in a dialysis cup, which was then immersed in 10 mL of PBS containing 1% w/v TW20 to maintain sink conditions. The system was continuously stirred for 120 hours. At predetermined time intervals (1, 3, 5, 8, 24, 48, 72, 96, and 120 hours), 1 mL aliquots were withdrawn for analysis. The concentration of Cur in the samples was quantified by measuring absorbance at 425 nm using a microplate reader. The formulation prepared with an organic phase containing 3 mg mL^−1^ of Cur, at an O/W mixing ratio of 1 : 3 (SLN-F1), exhibited approximately 50% drug release at 8 hours and reached a cumulative release of 102.76 ± 0.22% at 120 hours. In contrast, the formulation containing 5 mg mL^−1^ of Cur showed approximately 40% drug release at 48 hours. At 120 hours, the sample with an O/W ratio of 1 : 3 (SLN-F3) exhibited a cumulative drug release of 79.70 ± 0.64%, while the sample with the sample with a 1 : 5 ratio (SLN-F4) achieved a release of 109.05 ± 0.84%. All formulations exhibited a sustained drug release profile, characterized by an initial burst release phase followed by a sustained release phase. This release pattern is consistent with previous literature, which reports that over 40% of the drug is released within the first 24 hours. However, this does not indicate particle instability.^33^ Instead, it is attributed to the inherent characteristics of SLNs, where drug release occurs through the gradual erosion and degradation of the solid lipid matrix. The initial burst release is likely due to drug molecules adsorbed on the nanoparticle surface or localized within the outer lipid layer, while the sustained release phase is governed by the gradual diffusion of the encapsulated drug from the lipid core.^46^ SLNs with sustained drug release over a long period may degrade before the drug is completely released due to first-pass hepatic metabolism. The SLNs we developed will be incorporated into microneedle formulations for transdermal delivery in future studies. Transdermal delivery of Cur-SLNs using microneedles will not only bypass first-pass hepatic metabolism but also be a promising formulation for treating localized skin diseases.^47^

### 3.5 Stability test of Cur-SLNs

**Fig. 7:**
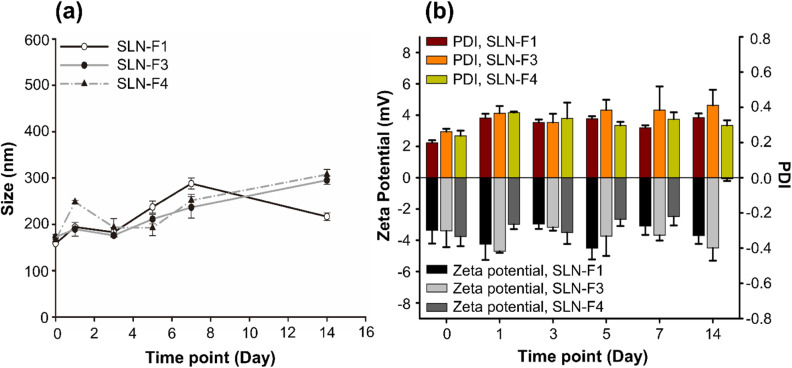
(a) Particle size change to check the storage stability of SLN. (b) The changes in zeta potential and PDI of SLN during storage stability testing. It was observed after diluting the particles in PBS maintained at 37 °C. Results are reported as mean ± SD, *n* = 3.

*In vitro* stability studies were conducted to evaluate the colloidal stability of Cur-SLNs. Poor colloidal stability prior to *in vivo* administration can lead to particle aggregation, which may negatively affect drug bioavailability and release kinetics, ultimately compromising therapeutic efficacy. To assess the stability of Cur-SLNs, changes in particle size were monitored in PBS (pH 7.4) over 14 days. SLN-F1, which was prepared using an organic phase containing 3 mg mL^−1^ of Cur and an O/W mixing ratio 1 : 3, exhibited a particle size increase of approximately 58.3 nm in PBS. In comparison, SLNs formulated with an organic phase containing 5 mg mL^−1^ of Cur at O/W ratios of 1 : 3 (SLN-F3) and 1 : 5 (SLN-F4) showed larger particle size increases of 122.8 nm and 141.2 nm, respectively (Fig. 7a). After 14 days, the PDI value increased the most in the SLN-F3 formulation, reaching approximately 0.15, while in the other formulations, it increased slightly to 0.08 in SLN-F1 and 0.06 in SLN-F4 (Fig. 7b). No significant differences were observed in the zeta potential among the formulations.

In conclusion, SLN-F1 demonstrated the highest stability, exhibiting minimal changes in particle characteristics. This finding is consistent with previous literature, which suggests that stability decreases as drug content increases under the same conditions.^40^ These results also indicate that higher surfactant concentrations may be necessary to maintain colloidal stability as drug loading increases. The surfactant layer formed on the surface of cetyl palmitate plays a crucial role in enhancing long-term storage stability.^48^ Furthermore, the incorporation of PEGylated lipids has been widely reported to prevent particle aggregation and improve the storage stability of nanoparticle formulations.^49^

Therefore, the SLNs developed in this study exhibited stability through PEGylation despite their low zeta potential values, demonstrating properties favorable for cellular delivery. Based on these findings, future studies will focus on evaluating the *in vivo* drug delivery efficiency of these SLNs. Additionally, further efforts will be directed toward developing them as transdermal or injectable formulations rather than oral administration to enhance drug delivery efficiency.

## 4. Conclusion

We successfully synthesized Cur-SLNs with high encapsulation efficiency and stability under controlled temperature conditions using a microfluidic machine. This synthesis method offers the advantage of loading various drugs and allows for the production of nanoparticles with desired characteristics by selecting the appropriate lipids and surfactants based on drug concentration. Additionally, using a microfluidic machine to manufacture nanoparticles ensures high reproducibility and suggests the potential for large-scale production through scale-up from laboratory to industrial scale. FT-IR and DSC analyses confirmed that Cur was successfully encapsulated within the SLNs, and the *in vitro* Cur release experiment showed approximately 80% cumulative drug release over 120 hours. In the stability test of Cur-SLNs, the particle size increased by 58.3 nm over 14 days, and this increases was attributed to the surfactant layer formed by cetyl palmitate used in the synthesis process, contributing to the long-term storage stability of Cur-SLNs. This study is meaningful in establishing a system that can efficiently deliver poorly soluble drug (or Cur) by synthesizing SLNs with these characteristics and applying them in clinical settings.

## Data availability

The authors report that the results of this study are available within the manuscript.

## Conflicts of interest

The authors declare that they have no known competing financial interests or personal relationships that could have appeared to influence the work reported in this paper.
